# Effects of different exercise intensities on prefrontal activity during a dual task

**DOI:** 10.1038/s41598-022-17172-5

**Published:** 2022-07-29

**Authors:** Daisuke Kimura, Takayuki Hosokawa, Takuya Ujikawa, Tomotaka Ito

**Affiliations:** 1 Graduate School of Health Science and Technology, Kawasaki University of Medical Welfare, 288 Matsushima, Kurashiki, Okayama 701-0193 Japan; 2 Department of Physical Therapy, Faculty of Rehabilitation, Kawasaki University of Medical Welfare, 288 Matsushima, Kurashiki, Okayama 701-0193 Japan; 3 Department of Orthoptics, Faculty of Rehabilitation, Kawasaki University of Medical Welfare, Kurashiki, Japan

**Keywords:** Premotor cortex, Rehabilitation

## Abstract

The effects of physical exercise on cognitive tasks have been investigated. However, it is unclear how different exercise intensities affect the neural activity. In this study, we investigated the neural activity in the prefrontal cortex (PFC) by varying the exercise intensity while participants performed a dual task (DT). Twenty healthy young adults performed serial subtraction while driving a cycle ergometer. Exercise intensity was set to one of three levels: low, moderate, or high intensity. We did not find any significant change in PFC activity during DT under either the control (no exercise) or low-intensity conditions. In contrast, we observed a significant increase in PFC activity during DT under moderate- and high-intensity conditions. In addition, we observed complex hemodynamics after DT. PFC activity decreased from baseline after DT under the control condition, while it increased under the low-intensity condition. PFC activity remained higher than the baseline level after DT under the moderate-intensity condition but returned to baseline under the high-intensity condition. The results suggest that moderate-intensity exercise with a cognitive load effectively increases PFC activity, and low-intensity exercise may increase PFC activity when combined with a cognitive load.

## Introduction

In daily life, physical and cognitive activities often occur simultaneously (e.g., we can think while walking) rather than singly. To understand the physiological states during simultaneous exercises, a dual-task paradigm has been used. Indeed, a dual task is widely used in clinical practice and research fields as an assessment method to predict the risk of falls and gain better understanding of frontal lobe functions, including executive functions^1–7^. Functional near-infrared spectroscopy (fNIRS) is often used to investigate brain activity during a dual task since it can quantify the hemodynamics in the brain while a participant performs a physical exercise^8^.

The activity in the prefrontal cortex (PFC) is modulated by the physical as well as the cognitive load^9,10^. It has been reported that as the intensity of physical exercise increases, the concentration of oxy-Hb in PFC increases to some extent and then decreases^11–17^. As a result, the relationship between the exercise intensity and oxy-Hb concentration in PFC shows an inverted-U function. High intensities of exercise are predicted to interfere with cognitive processes through the increase in neural noise, elevation of the arousal level, or down-regulation of PFC activity^17^. A similar phenomenon is observed for cognitive load, where a more significant load of cognitive tasks also produces the inverted-U function relationship between cognitive load and prefrontal activity^18,19^. In a dual task, where both physical and cognitive loads are charged simultaneously, the hemodynamics in PFC are assumed to become more complex due to the increase or decrease in the amount of mutual loads^20–26^.

In a dual task, factors such as fatigue, the intensity of the exercise, and the complexity of the task cause the inhibition and facilitation of brain functions, which is known as cognitive-motor interference^27^. The interference induces a change in PFC activity. The simultaneous performance of cognitive and physical tasks requires greater recruitment of oxy-Hb to PFC^22,24,28^. Mandrick et al.^22^ have reported that in a dual task, where a computational task was introduced to an isometric grasping contraction task, a higher concentration of oxy-Hb was observed in PCF. Moreover, the performance of the computational task significantly deteriorated, whereas the force variability significantly increased at 30% maximum voluntary contraction (MVC) compared with 15% MVC. Mirelman et al.^24^ reported that when a cognitive task (serial subtraction) was introduced in addition to a physical task (walking), the walking speed decreased compared with a single task (i.e., walking only). Other studies also showed that motor performance tends to deteriorate during dual tasks^21,28–33^.

On the other hand, it is also reported that the cognitive task performance can be improved by exercise^34–37^. A meta-analytic study showed that an acute, intermediate-intensity exercise improves the performance of working memory tasks probably because of the increased arousal level^36^. Additionally, it has been reported that even a low-intensity exercise and walking can improve cognitive functions during and after the exercise^38,39^.

It has been reported that different intensities of physical load modulate PFC activity during exercise, and the frontal lobe functions improve after the exercise, which indicates that the changes in frontal lobe activity caused by an exercise are sustained even in the postexercise period^35,38^. Therefore, it is likely that the dual task affects brain activity not only during but also after a physical exercise. However, it is unclear how different exercise intensities affect PFC activity during a dual task. If the sustained effects of a dual task on PFC activity are confirmed, it may lead to the development of new exercise programs to improve PFC functions. In this study, we investigated the effects of exercise intensity during a dual task on the hemodynamics of oxy-Hb in the PFC. In rehabilitation, therapists occasionally use dual tasks for patients or the elderly to improve their cognitive functions such as attention and working memory, in which PFC plays an important role^40–44^. In this study, we aimed to determine the exercise intensity that effectively activated the PFC in a dual task. If a low-intensity exercise with a cognitive load activated the PFC, it would benefit patients or elderly people unable to perform high-intensity exercises.

## Methods

### Participants

Twenty healthy young adults (mean ± SD: age, 20.7 ± 3.7 years; height, 169.0 ± 4.5 cm; weight, 59.8 ± 4.5 kg) were studied. None of the participants were athletes. Only male participants were recruited to avoid gender differences with respect to cortical oxygenation responses^45,46^. They had no underlying orthopedic, neurological, and cardiovascular disorders. Each participant provided written informed consent before enrolling in this study. The study conformed to the principles of the Declaration of Helsinki and the protocol of Kawasaki University of Medical Welfare, and the Research Ethics Committee approved the study (approval number 19-060).

### Procedure

All participants were required to visit the lab twice on different days. On their first visit, a cardiopulmonary exercise test was performed using an expiratory gas analyzer (POWER METS AT-1100A, ANIMA, Japan) to determine the intensity of exercise, and the maximum oxygen uptake (VO_2_peak) was calculated for each participant. A bicycle ergometer (Strength Ergo8, MITSUBISHI ELECTRIC, Japan) was used during the cardiopulmonary exercise test, and the participants kept the pedal rotation rate at 60 rpm under the incremental load condition of 20 w/min^47–50^. The endpoint of the incremental exercise was determined when either of the following three conditions occurred: (1) cyanosis or pallor, (2) a pedal rotation rate below 55 rpm for more than 3 s, and (3) an appeal to end the exercise by the participant. Participants had a cool-down exercise after the end of the incremental exercise.

On their second visit, 2–7 days after the first visit, the participants performed a dual task where they serially subtracted three from 100 while carrying out pedal rotations on the ergometer. To make the dual task easier, we employed a serial subtraction of three rather than seven because, in the pretest, some participants complained of the difficulty of performing a serial subtraction of seven, which has been used in other studies^51–53^, under the high-intensity load condition. We had four different conditions in the dual task according to the exercise intensity: (a) pedaling exercise at a low-intensity (2) pedaling exercise at a moderate-intensity, (3) pedaling exercise at a high-intensity, and (4) sitting still on the ergometer (i.e., control condition). The order of these four conditions was randomized among the participants. Each condition consisted of the following four phases: resting (baseline), pedaling (single motor task before dual task), dual task (pedaling and serial subtraction), and pedaling (single motor task after dual task). While the participants performed these exercises, we measured PFC activity by fNIRS and oxygen uptake using an expiratory gas analyzer. The duration of the experiment was approximately 60 min, including breaks.

### Assessment of oxygen uptake during pedaling exercise

The oxygen uptake during pedaling exercise was assessed using the expiratory gas analyzer. In the Guidelines for Rehabilitation in Patients with Cardiovascular Disease^54^, the low-intensity load is indicated as 20–40% for the VO_2_peak, the moderate-intensity load as 40–60%, and the high-intensity load as 60–70%. In this study, we used the following criteria: 23% VO_2_peak for the low-intensity load, 40% VO_2_peak for the medium-intensity load, and 60% VO_2_peak for the high-intensity load. The intensity of exercise was calculated for each participant according to the VO_2_peak of each participant. Intensity levels were controlled by changing the pedal load. When the pedaling exercise started, the participants were required to reach the designated pedaling speed (50 cycles/min) and maintain the speed. The pedaling speed was displayed on the ergometer during the task. The pedaling exercise consisted of 1 min of rest, 2 min of warm-up, 3 min of pedaling with a load (motor task 1: M1), 2 min of the dual task (DT: motor task + cognitive task), 1 min of the motor task (motor task 2: M2), and 1 min of cool-down exercise. The warm-up duration was determined so that the hemodynamics became stable. All participants performed each condition (low, moderate, high intensity, and control) in a randomized order.

### Assessment of frontal lobe activity

**Figure 1:**
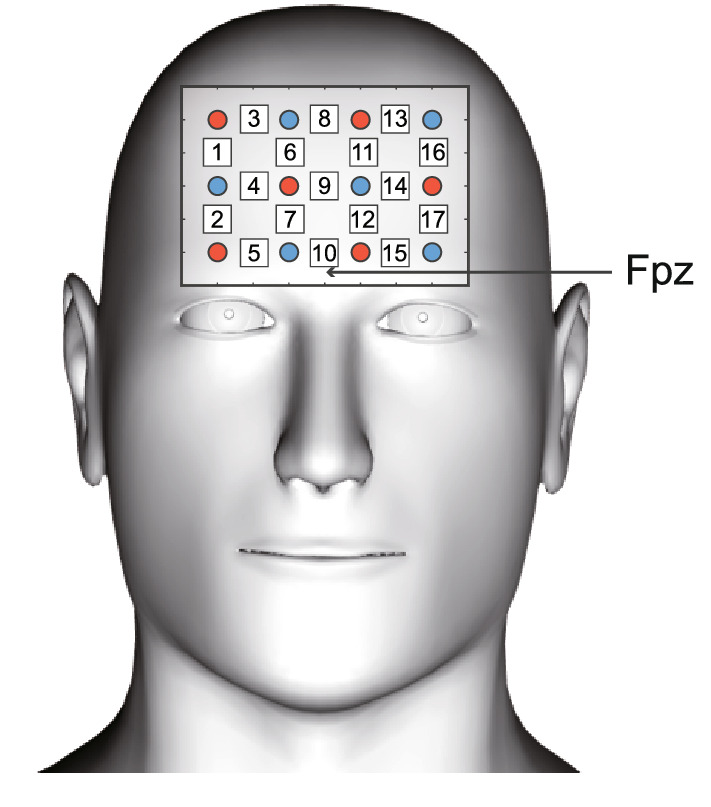
NIRS channel layout. The red circles indicate the emitter probes, and the blue circles indicate the detector probes. Each number indicates the channel number.

We measured the PFC activity using an fNIRS system (SpectratechOEG-17APD, Spectratech Inc., Japan) with 17 channels (3 × 4 probe arrangement). PFC was selected as the measurement location because it is related to attention and higher cognitive functions^55,56^. We measured the oxy-Hb concentration in the right and left PFC regions by fixing the center-bottom channel (channel 10) at Fpz according to the international 10–10 method (Fig. 1). Both oxy-Hb and deoxy-Hb concentrations were measured as indicators of oxy-Hb concentration changes. Since it was previously reported that head tilts affect fNIRS measurement^57^, the participant was verbally instructed to minimize head movement during the dual task, and one of the experimenters held down a participant's shoulders to prevent the participant's trunk from swaying while the participant was driving the ergometer. We covered the probe set with an elastic band to prevent it from shifting on the head and prevent light from entering through gaps. Each participant was fitted with the probe set and instructed to rest for a while. The fNIRS measurement was initiated after the hemodynamics became stable.

### Data analysis

We analyzed the expiratory gas data in an epoch of 30 s before the end of each of the three periods M1, DT, and M2. Each participant completed the four different loads of pedaling exercise during the experiment. Since the fNIRS measurements and ergometer were manually synchronized with a hand button, there was a variance of 3–5 s in the data length. Therefore, the data available for the analyses were 170 s for M1, 115 s for DT, and 49 s for M2. We used the data in these entire intervals to analyze the fNIRS data in DT and MT. For the analysis of M1, the hemodynamics in the initial period was not stable owing to the effect of body circulations in high-intensity load; thus, we used the data after an interval of 70 s after the start of the M1 period.

The expiratory gas was measured at a sampling rate of 100 Hz. We carried out breath-by-breath analysis to measure the respiratory gas exchange, thus analyzing the oxygen uptake at each breath. The mean oxygen uptake level in each task period was used as the index. We analyzed the oxygen uptake level by repeated two-way analysis of variance (with period and exercise intensity as the factors), followed by the Tukey test (Tukey–Kramer method) for multiple comparisons.

The fNIRS signals were measured at a sampling rate of 12.2 Hz. The raw data were processed with a 0.01–0.3 Hz band-pass filter to remove physiologically irrelevant effects. The mean of the fNIRS signal intensities during the rest interval was subtracted from that during each task interval. Then, the signal intensities during each interval were converted to Z-scores by dividing them by the standard deviation of the signal intensity during the rest interval^58–61^. The channels whose mean signal intensities exceeded the mean ± two standard deviations (SD) of all channel data were excluded from the analysis. To observe the time-series changes of hemodynamics, each task period was divided into two parts, the first half and the second half of the period. (Fig. 1). We selected the channels with significant changes in oxy-Hb concentration in the task periods compared with the rest period and used the mean values of those channels as the index of oxy-Hb in each task period. For statistical processing, we compared the values in DT, M1, and M2 with those in the rest period and the values in M1 and M2 with those in DT using paired t-test.

The significance level was set at 0.05 (two-tailed). All statistical analyses were performed using IBM SPSS Statistics 25 (IBM SPSS Statistics Inc., Tokyo, Japan).

### Ethics approval and consent to participate

All participants provided written informed consent and the study was approved by the Kawasaki University of Medical Welfare (No.19-060).

### Consent for publication

Written informed consent for publication was obtained from all the participants.

## Results

### Oxygen uptake during the tasks

The mean oxygen uptake levels for 30 s before the end of M1, DT, and M2 for each exercise intensity are shown in Table 1. The mean maximal oxygen uptake level across all participants was 45.2 ± 8.0 ml/min/kg. Repeated two-way analysis of variance showed the significant main effects of both the task and exercise intensity factors [F_2,38_ = 9.131, *p* = .001; F_3,57_ = 364.075, *p* < .001]. A significant interaction was also observed [F_6,114_ = 12.286, *p* < .001]. The simple main effect of the exercise intensity factor was significant under all task periods [M1: F_3,57_ = 306.788, *p* < .001; DT: F_3,57_ = 371.450, *p* < .001; M2: F_3,57_ = 356.942, *p* < .001]. Furthermore, the simple main effect of the task factor was significant under the high-intensity exercise condition [F_2,38_ = 30.369, *p* < .001]. The post-hoc test for the exercise intensity factor (Bonferroni corrected) showed that the oxygen uptake level significantly increased in the order of no-intensity (control condition), low-intensity, moderate-intensity, and high-intensity conditions in all task periods (i.e., M1, DT, and M2). Moreover, the post-hoc test for the task factor showed that the oxygen uptake level was significantly lower in M1 than in DT and M2 under the high-intensity condition.

**Table 1:** Mean oxygen uptake levels under different exercise intensity conditions.

	Motor task1 Mean (SD)	Dual task Mean (SD)	Motor task2 Mean (SD) (ml/min/kg)
Control	4.7 (1.7)	4.6 (1.9)	4.6 (2.0)
Low-intensity	11.1 (1.8)	11.0 (1.9)	10.6 (1.8)
Moderate-intensity	20.4 (3.4)	20.6 (3.2)	20.5 (3.1)
High-intensity	28.2 (4.8)*,**	30.1 (5.3)	30.1 (5.1)

*Significant difference between M1 and DT under the high-intensity condition in post-hoc analysis.

**Significant difference between M1 and M2 under the high-intensity condition in post-hoc analysis.

### PFC activity in each channel during each task

**Figure 2:**
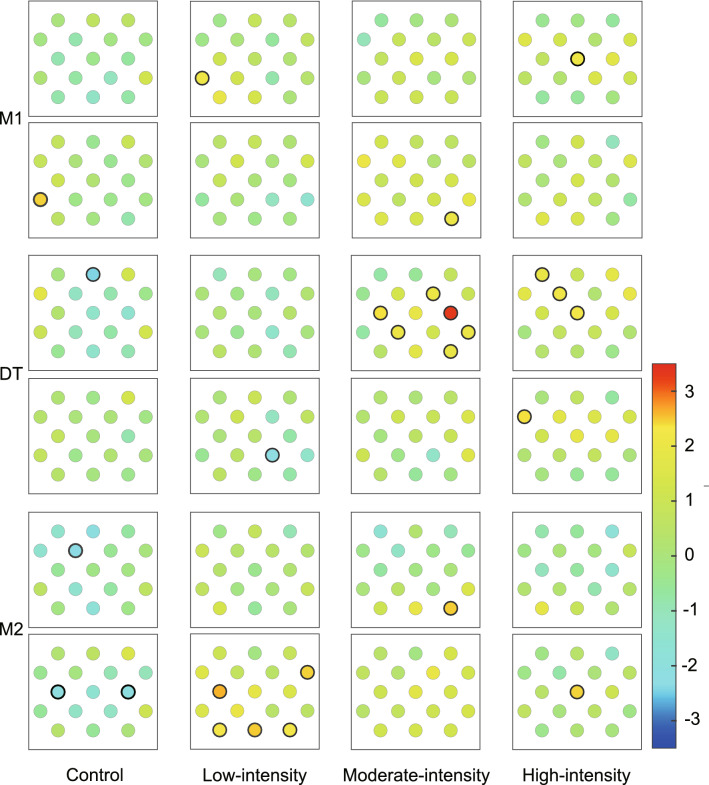
Topographical maps of PFC activation under control, low-intensity, moderate-intensity, and high-intensity conditions. The circles represent channels, the locations of which correspond to the channels shown in Fig. 1. The color bar indicates the t-value, which indicates the increase or decrease in oxy-Hb concentration from the resting period. The bluish color indicates the decrease in oxy-Hb concentration during the task (M1, DT, and M2) relative to the resting period, whereas the reddish color indicates the increase. The channel with a significant difference is outlined with a thicker black circle (*p* < .05). We separately analyzed the concentration of oxy-Hb during the first and second halves of M1, DT, and M2. The upper panels of each of the periods M1, DT, and M2 in the figure show the results of the first half and the lower panels show those of the second half.

We compared the oxy-Hb concentration under each task condition with that in the resting period for each channel (t-test). We found a significant difference in channels 2, 4, 6, 8, and 14 under the control condition; channels 2, 4, 5, 10, 12, 15, 16, and 17 under the low-intensity condition; channels 4, 7, 11, 14, 15, and 17 under the moderate-intensity condition; and channels 1, 3, 6, and 9 under the high-intensity condition (Fig. 2).

### Effects of exercise intensity on PFC activity

**Figure 3:**
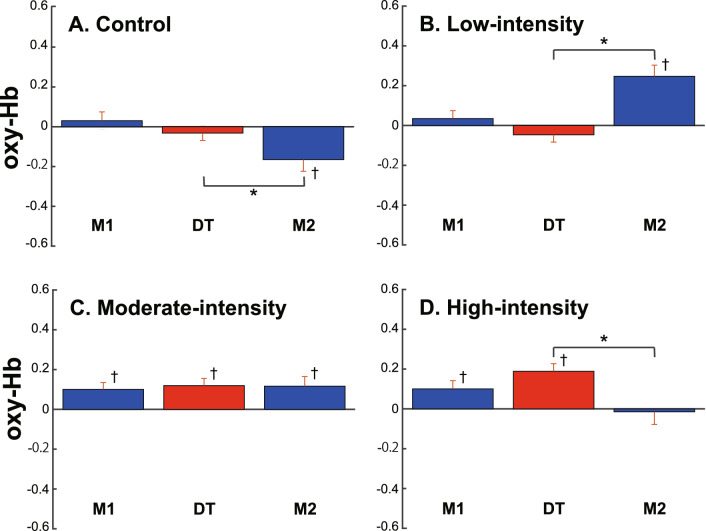
Normalized mean oxy-Hb concentration during the task for each exercise intensity. The oxy-Hb concentration was normalized by that in the resting period and averaged for each exercise intensity. Mean ± SEM. *A significant difference between the task conditions (*p* < .05). ^†^A significant difference from under the rest condition (*p* < .05).

We show in Fig. 3 the mean oxy-Hb concentration in the channels where a significant difference was found. We pooled the data from those channels for each exercise intensity. We first analyzed whether the oxy-Hb concentrations in the task periods were significantly different from that in the resting period. In M1 and DT, we found significantly higher oxy-Hb concentrations under the moderate-intensity and high-intensity conditions, but not under either the control or low-intensity condition [moderate intensity: t_109_ = − 3. 227, *p* = .002, d = .46, power = .95, t_109_ = − 3.452, *p* = .001, d = .51, power = .97; high intensity: t_67_ = − 2.136, *p* = .036, d = .35, power = .76, t_67_ = − 4.293, *p* < .001, d = .72, power = .99; control: t_91_ = − .50, *p* = .62, d = .92, power = 1.0, t_91_ = .94, *p* = .35, d = .14, power = .67; low intensity: t_126_ = − 1.27, *p* = .21, d = .17, power = .74, t_126_ = .64, *p* = .53, d = .53, power = .70]. In M2, we found mixed results depending on the intensity of exercise. The mean oxy-Hb concentration in M2 was significantly lower than the baseline under the control condition [t_91_ = 2.746, *p* = .007, d = .42, power = .89] but was significantly higher under the low-intensity condition [t_126_ = − 4.625, *p* < .001, d = .49, power = .98]. Under the moderate-intensity condition, it was significantly higher than the baseline [t_109_ = − 2.897, *p* = .005, d = .36, power = .81] but comparable to that in DT. Under the high-intensity condition, it returned to the baseline and was significantly lower than that in DT.

We found a significant difference in PFC activity between M1 and DT both under the low-intensity condition [t_126_ = 2.718, *p* = .007, d = .19, power = .36] and the high-intensity condition [t_67_ = − 2.830, *p* = .006, d = .27, power = .28]. However, the effects of size and power were both small and not significantly different from the baseline under either condition. Moreover, because the whole-body oxygen uptake had not reached the stable state in M1 under the high-intensity condition, it was difficult to compare the oxy-Hb concentration between M1 and DT. Therefore, we did not consider that these results reflect a valid difference.

We then compared the mean oxy-Hb concentration in DT with that in M2. We found a significant increase in oxy-Hb concentration from DT to M2 under the low-intensity condition [t_126_ = − 6.458, *p* < .001 d = .55, power = .99]. On the other hand, we found a significant decrease in oxy-Hb concentration from DT to M2 in the control and high-intensity conditions (t_91_ = 2.538, *p* = .013, d = .42, power = .93; t_61_ = 3.195, *p* = .002, d = .47, power = .67).

## Discussion

In this study, we aimed to clarify the effects of exercise intensity on PFC activity during the dual task. To find how PFC activity changes over time depending on different intensities of exercises during the dual task, we first determined the exercise intensities for each participant based on their maximal oxygen uptake. The mean value of the peak oxygen uptake (45.2 ± 8.0 ml/min/kg) was similar to that in a previous study^62^, which showed that the peak oxygen uptake in males between the ages of 20 and 29 was 47.2 ± 7.9 ml/min/kg. The oxygen uptake values were stable during the three periods (M1, DT, and M2) under the control, low-intensity, and moderate-intensity conditions. On the other hand, they were significantly higher in DT and M2 than in M1 under the high-intensity condition. The high-intensity condition (60% of the VO_2_peak) was beyond the anaerobic threshold (AT), which is 46–56% of the VO_2_peak^63–65^. Since it may take more than 2 min for the oxygen uptake to reach the steady state at load intensities higher than AT^66^, it is possible that the oxygen uptake had not reached the steady state during the 2-min warm-up under the high-intensity condition. Nevertheless, there was no significant difference in the oxygen uptake values between DT and M2 under the high-intensity condition. From these facts, it is plausible that the participants' body circulation of oxygen had reached a steady state except for M1 under the high-intensity condition.

We found different PFC activities depending on the task periods and the exercise intensities: PFC activity was significantly higher in M1 and DT under moderate-intensity and high-intensity conditions, but not under control or low-intensity conditions. This result indicates that a threshold of the exercise intensity that facilitates PFC activity may exist between low-intensity (20% of VO_2_peak) and moderate-intensity (40% of VO_2_peak) loads. This result is consistent with previous studies showing that PFC activity increases under moderate- and high-intensity-loading conditions^35^. The exercise under the low-intensity condition was comparable to 3 METS of exercise such as walking^67^. Since previous studies showed that PFC activity increased during walking^24,39^, we expected that PFC activity would increase under the low-intensity condition. However, PFC activity did not significantly increase in M1 or DT under the low-intensity condition. The reason for this discrepancy is unclear, but it may be due to the difference in the types of exercise (i.e., walking or driving an ergometer).

Interestingly, PFC activity after the dual task (M2) was differed depending on the exercise intensity. PFC activity remained high in M2 under the moderate-intensity condition, but it returned to the baseline level in M2 under the high-intensity condition. Since the interference often occurs when the task is difficult^68^, the interference may cause the decrease in PFC activity after the dual task under the high-intensity condition. Thus, the result of this study indicates that the interference in the dual task may occur between 40 and 60% motor intensities. Moreover, PFC activity decreased after the dual task (M2) under the control condition but increased under the low-intensity condition. These results suggest that there are delayed effects on PFC activity in the dual task depending on the exercise intensity. Many studies examined the effects of aerobic exercise on cognitive performance^38,69–80^. In those studies, cognitive functions were measured at least 1 to 15 min after the exercise, and most of them found the improvement of cognitive functions^38,71,73,77–80^. Considering that there is a positive relationship between cognitive function and PFC activity^38,74,80^, it is highly likely that PFC activity increases between 1 and 15 min after exercise, and that there are delayed effects of exercise on PFC activity, although the mechanism of the delayed effects is unclear. Thus, the increase in PFC activity after the dual task under the low-intensity condition may reflect such delayed effects. In summary, the results of this study suggest that PFC functions increase after a dual task with low-intensity exercise as well as during moderate- to high-intensity exercise.

As a limitation of this study, it is impossible for us to know the strategies the participants used because there were no performance measures for the cognitive task. Theoretical models for performance in dual tasks have been proposed^80,81^, and it has been suggested that participants may use different strategies according to the difficulty level of the task. Since the participants were required to maintain the designated pedaling speed (50 cycles/min), they might have used a strategy to adjust the cognitive load (i.e., slowing down the subtraction) when the dual task was difficult for them. Thus, we cannot rule out the possibility that the participants might have used different strategies according to the exercise intensity.

Another limitation is that it was impossible to confirm whether the participants correctly performed a serial subtraction because they were asked to do the calculation mentally. Although in a study by Ohsugi et al.^82^, the participants were asked to orally answer the numbers in a serial subtraction task, we asked the participants not to answer orally because an utterance would affect fNIRS recordings. Despite this limitation, we think that the participants correctly performed the task because the serial subtraction was easy in this study (subtraction of 3, instead of 7, see Methods) and the cognitive load affected the PFC activity in the periods during and after the dual task (i.e., DT and M2).

There is also a limitation in generalizing the results of this study because we did not pre-calculate the ideal sample size to ensure an adequate power to detect statistical significance, and the sample size was small. In addition, because the study was conducted with only male participants, we cannot rule out sex differences.

## Conclusions

The PFC activity was measured using fNIRS when the participants were driving the ergometer at different intensities of exercise and simultaneously performing a serial subtraction in their mind. We found different hemodynamics in PFC depending on the exercise intensity. Under the low-intensity condition, the PFC activity increased only after the dual task. Under the moderate-intensity condition, it increased during the first exercise task (M1) and remained high during and after the dual task (DT and M2). Under the high-intensity condition, it increased during the dual task but returned to the baseline after the dual task, probably due to the interference effects from the high-intensity exercise. From the practical point of view, it may be proposed that, based on the result that PFC activity increased after the dual task with the low-intensity exercise, the elderly and individuals who are unable to perform high-intensity exercises may benefit from performing a low-intensity exercise combined with a cognitive task.

## Data Availability

The datasets used or analyzed during the current study are available from the corresponding author upon reasonable request.

## Abbreviations

fNIRS: Functional near-infrared spectroscopy
PFC: Prefrontal cortex
oxy-Hb: Oxygenated hemoglobin
